# Association between systemic inflammation biomarkers and cancer cachexia in patients with gastric cancer: a cross-sectional study

**DOI:** 10.3389/fnut.2026.1737375

**Published:** 2026-02-11

**Authors:** Yan Huang, Jinxin Zhang, Yan Ge, Chen Wang, Xiuhua Wang, Junbo Zuo

**Affiliations:** 1 Department of Clinical Nutrition, The Affiliated People’s Hospital of Jiangsu University, Zhenjiang, China; 2 Department of Cardiovascular Medicine, The Affiliated People’s Hospital of Jiangsu University, Zhenjiang, China; 3 Department of General Surgery, The Affiliated People’s Hospital of Jiangsu University, Zhenjiang, China

**Keywords:** advanced lung cancer inflammation index, biomarkers, cancer cachexia, gastric cancer, systemic inflammation

## Abstract

**Background:**

Systemic inflammation is regarded as a key driver of cancer cachexia. This study aimed to explore the relationship between systemic inflammatory biomarkers and cancer cachexia and compare their predictive capabilities in patients with gastric cancer (GC).

**Methods:**

Cancer cachexia was diagnosed according to Asian Working Group for Cachexia (AWGC) criteria. Logistic regression analyses were utilized to evaluate the association between systemic inflammation biomarkers and cancer cachexia. The receiver operating characteristic curve was used to assess the predictive performance of these biomarkers in identifying cancer cachexia.

**Results:**

A total of 440 patients were included in this study. Among them, 167 patients (37.95%) were diagnosed with cachexia. In GC patients with cachexia, systemic inflammation biomarkers including neutrophil-to-lymphocyte ratio (NLR), systemic immune-inflammation index (SII), systemic inflammatory response index (SIRI), aggregate index of systemic inflammation (AISI), inflammatory prognostic index (IPI), and inflammatory burden index (IBI) were significantly elevated, while the advanced lung cancer inflammation index (ALI) was significantly decreased (all *p* < 0.001). After adjusting for potential confounding variables, all these systemic inflammation biomarkers were significantly associated with cancer cachexia (all *p* < 0.001). According to the Delong test, ALI showed the highest AUC (AUC = 0.746, 95%: 0.698–0.794) compared with the other systemic inflammation biomarkers (all *p* < 0.05).

**Conclusion:**

Our findings demonstrated that NLR and NLR-related systemic inflammatory biomarkers (SII, SIRI, AISI, IPI, IBI, and ALI) were significantly associated with cancer cachexia. Among these biomarkers, ALI exhibited the highest efficacy in identifying GC patients with cancer cachexia.

## 1 Introduction

Cachexia is highly prevalent among patients with cancer, especially those with advanced malignant tumors. Cancer cachexia is a multifactorial syndrome characterized by significant weight loss and skeletal muscle wasting, with a prevalence ranging from 11 to 74% (1, 2). This syndrome can result in progressive functional impairment, treatment-related complications, a decreased quality of life, and an increased risk of cancer-related mortality in cancer patients (3). Additionally, cachexia accounts for 20% of deaths related to cancer directly (4). Therefore, the timely identification and assessment of cachexia are essential for preventing adverse outcomes in cancer patients.

Cachexia is driven by a complex interplay of diminished food intake and metabolic alterations, such as increased energy expenditure, excessive catabolism, and systemic inflammation (3). Systemic inflammation is regarded as a key driver of cancer cachexia (5). According to the European Society for Clinical Nutrition and Metabolism (ESPEN) guidelines on definitions and terminology of clinical nutrition, cachexia was defined as a synonym of disease-related malnutrition with inflammation (6). It is known that inflammation not only contributes to the development of cachexia but also plays a pivotal role at multiple stages of cancer. Systemic inflammation associated with cancer is induced by a multitude of factors, including the release of pro-inflammatory cytokines and other inflammatory mediators by tumor cells, as well as the secretion of cytokines and chemokines by activated immune cells (7). It has been observed that patients with cancer cachexia exhibit elevated circulating levels of pro-inflammatory cytokines with catabolic effects, such as interleukin-1 (IL-1), interleukin-6 (IL-6), interferon-*γ* (IFN-γ), and tumor necrosis factor-*α* (TNF-α) (8). C-reactive protein (CRP), an acute-phase protein, is mainly synthesized in hepatocytes when stimulated by pro-inflammatory cytokines like interleukin-1β (IL-1β) and IL-6 (9). An increased circulating level of CRP has also been shown to be associated with weight loss and skeletal muscle wasting in cancer patients (10). Recently, the Asian Working Group for Cachexia (AWGC) published the diagnostic criteria for cachexia in the Asian population. Notably, an elevated level of CRP, which serves as a key biomarker of systemic inflammation, has been incorporated into these diagnostic criteria (11).

In the clinical setting, routine hematological tests can effectively evaluate systemic inflammation, such as elevated pro-inflammatory markers like neutrophils and CRP levels, and decreased anti-inflammatory markers including lymphocytes and albumin levels. The neutrophil-to-lymphocyte ratio (NLR), which can be easily calculated from a routine complete blood count, is increasingly attracting attention as an indicator of systemic inflammation in various types of cancer (12, 13). In addition, NLR-related systemic inflammatory biomarkers that combine various inflammatory or anthropometric parameters, including systemic immune-inflammation index (SII) (14), systemic inflammatory response index (SIRI) (15), aggregate index of systemic inflammation (AISI) (16), inflammatory prognostic index (IPI) (17), inflammatory burden index (IBI) (18), and advanced lung cancer inflammation index (ALI) (19), have demonstrated significant involvement in cancer progression and patient outcomes.

Although systemic inflammation has been proven to play a crucial role in the development of cachexia, there is a lack of relevant research investigating the relationship between existing systemic inflammatory biomarkers and cancer cachexia. Therefore, the aim of this study was to explore the association between NLR-related systemic inflammatory biomarkers and cancer cachexia and to compare their predictive capabilities for identifying cachexia in patients with GC.

## 2 Materials and methods

### 2.1 Study population

**Figure 1:**
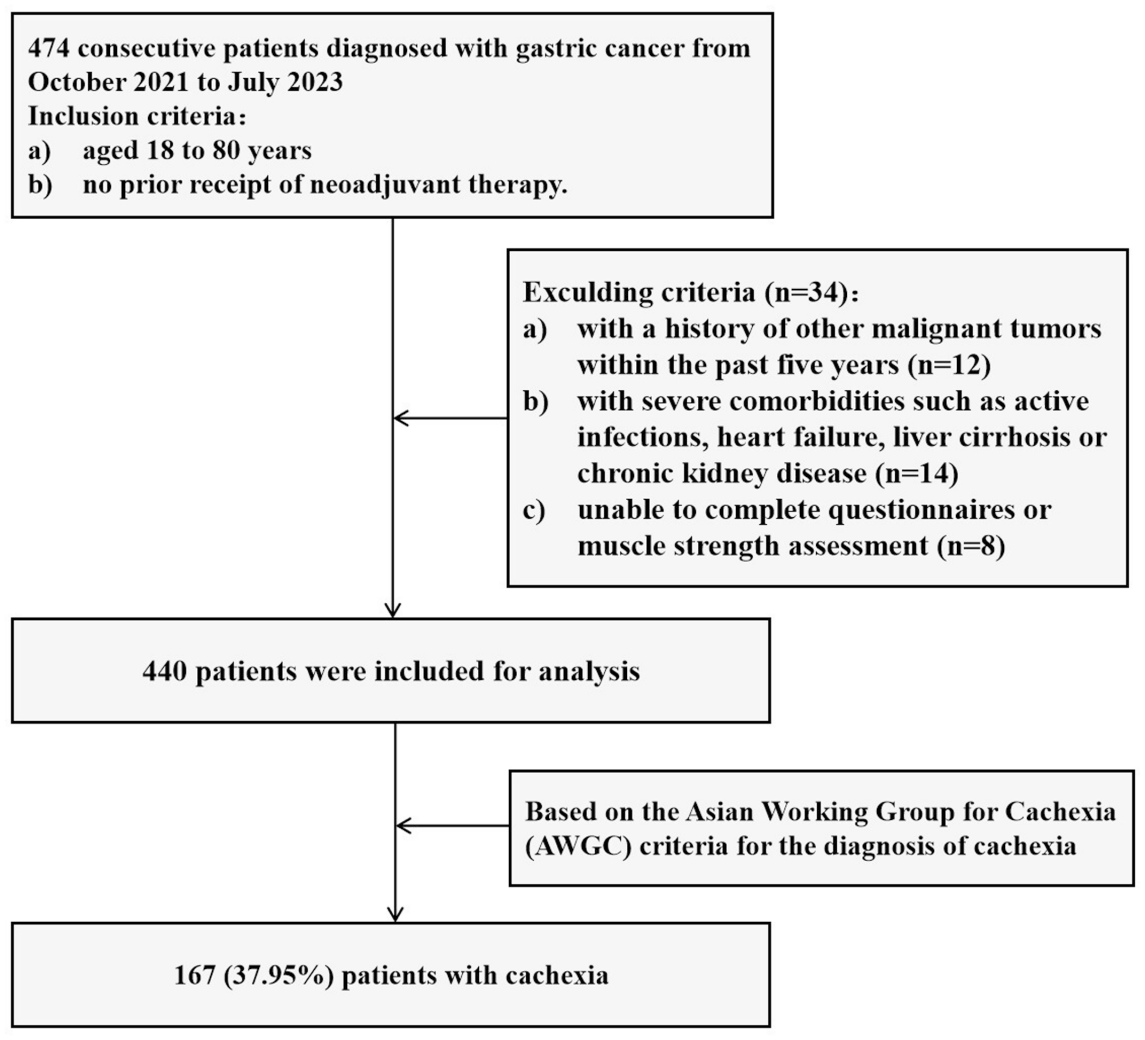
The flowchart outlining the inclusion and exclusion criteria for the study patients.

This cross-sectional study enrolled consecutive patients diagnosed with GC at The Affiliated People’s Hospital of Jiangsu University from October 2021 to July 2023. All patients aged 18 to 80 years who were diagnosed with GC and had not received neoadjuvant therapy were eligible for inclusion in this study. However, the following patients were excluded: (1) those with a history of other malignant tumors within the past 5 years; (2) those with severe comorbidities such as active infections, heart failure, liver cirrhosis or chronic kidney disease; and (3) those unable to complete questionnaires or muscle strength assessment. The detailed flowchart outlining the inclusion and exclusion criteria for the study patients was presented in Figure 1. All participants signed informed consent, agreeing to the collection and analysis of their data. The personal information of the participants was strictly confidential and was used only for research purposes, presented in an anonymous form. This research was conducted in accordance with the ethical guidelines outlined in the Declaration of Helsinki and obtained ethical approval from the Ethics Committee of the Affiliated People’s Hospital of Jiangsu University (No: KYW001-24).

### 2.2 Clinical and laboratory data collection

Routine blood tests were conducted in a fasting state within 24 h of admission. The clinical and laboratory data included sex, age, body mass index (BMI), percentage of weight loss (WL%), Nutritional Risk Screening-2002 (NRS-2002) score, Charlson Comorbidity Index (CCI) score, Eastern Cooperative Oncology Group performance status (ECOG PS), CRP level (mg/L), neutrophil count (10^9^/L), lymphocyte count (10^9^/L), platelet count (10^9^/L), monocyte count (10^9^/L), albumin (g/L), and hemoglobin (g/L). Patients with NRS-2002 score ≥ 3 were classified as being at risk of malnutrition (20). Hypoproteinemia was defined as the level of serum albumin < 35 g/L. Anemia was defined as the level of hemoglobin < 120 g/L in males and < 110 g/L in females. The formulas for calculating systemic inflammatory biomarkers were as follows: NLR = neutrophil / lymphocyte, SII = platelet × NLR, SIRI = monocyte × NLR, AISI = platelet × monocyte × NLR, IPI = CRP × NLR/albumin, IBI = CRP × NLR, and ALI = BMI × albumin/NLR.

### 2.3 Diagnosis of cancer cachexia

Cancer cachexia was diagnosed according to the AWGC criteria as follows: weight loss > 2% within 3–6 months or low BMI (< 21 kg/m^2^), along with one or more of the following conditions: (1) anorexia; (2) low handgrip strength (HGS); (3) elevated CRP (> 5 mg/L) (11). HGS was assessed employing a handheld dynamometer (EH101, Camry, Guangdong, China). Each hand was tested twice in an alternating order, and the maximum grip strength value was documented. Low HGS was categorized as < 28 kg for males and < 18 kg for females (21). Anorexia was evaluated using the anorexia symptom subscale of European Organization for Research and Treatment of Cancer Quality of Life Questionnaire version 3.0 (EORTC QLQ-C30). The scale includes response categories like “Not at all,” “A little,” “Quite a bit,” and “Very much.” Selecting “Not at all” reflects the absence of anorexia-related symptoms (22).

### 2.4 Statistical analysis

Continuous variables that followed a normal distribution (assessed by the Kolmogorov–Smirnov test) were expressed as mean ± standard deviation (SD) and compared using the Student’s t-test. Continuous variables that did not follow a normal distribution were presented as the median (first quartile, third quartile) and analyzed via the Mann–Whitney U test. Categorical data were shown as numbers (percentage) and examined using the chi-square test. Spearman’s correlation analysis was used to determine the correlations between WL% and systemic inflammatory biomarkers. To examine the independent relationship between systemic inflammatory biomarkers and cancer cachexia, multi-model logistic regression analysis was conducted by sequentially incorporating adjusted variables to develop three regression models. The crude model was adjusted for no covariate. Model I was adjusted for age, sex, and BMI. Model II was adjusted for age, sex, BMI, CCI, NRS-2002 score ≥ 3, ECOG PS, TNM stage, hypoproteinemia, and anemia. The values of systemic inflammatory biomarkers were standardized. Then, the relationships between the increase of these biomarkers per standard deviation (SD) and the presence of cancer cachexia were analyzed to obtain odds ratios (ORs) and 95% confidence intervals (CIs). Since BMI was a component of ALI, it was excluded from the multivariate analysis examining the association between ALI and the presence of cancer cachexia. The receiver operating characteristic (ROC) curve and the corresponding area under the curve (AUC) were used to assess the predictive performance of systemic inflammatory biomarkers in identifying cancer cachexia. The optimal cutoff point was determined by maximizing the Youden index. The DeLong test was applied to statistically compare the differences between ROC curves (23). Statistical analyses were carried out using SPSS version 25.0 (IBM Corp, Armonk, NY, USA) and MedCalc 20.03. A two-tailed *p*-value < 0.05 was considered statistically significant.

## 3 Results

### 3.1 Patient characteristics

A total of 474 patients with GC were screened, and 440 of them ultimately met the inclusion criteria (Figure 1). Among these patients, 316 (71.82%) were male and 124 (28.18%) were female, with a median age of 68 (62–72) years. There were 107 patients (24.32%) classified as TNM stage I, 84 patients (19.09%) as stage II, 189 cases (42.95%) as stage III, and 60 patients (13.64%) as stage IV. The demographic and clinical characteristics of these patients are summarized in Table 1. Using the NRS-2002 nutritional screening tool, a total of 286 patients (65%) were identified as being at risk for malnutrition. Further evaluation based on the AWGC diagnostic criteria revealed that 167 patients (37.95%) were classified as having cancer cachexia.

**Table 1:** Demographic and clinical characteristics of patients with gastric cancer.

Variables	Total (*n* = 440)	With cachexia (*n* = 167)	Without cachexia (*n* = 273)	*p*-value
Age, years	68.00 (62.00, 72.00)	69.00 (61.50, 72.50)	68.00 (62.00, 72.00)	0.424
Sex, *n* (%)				0.652
Male	316 (71.82)	122 (73.05)	194 (71.06)	
Female	124 (28.18)	45 (26.95)	79 (28.94)	
BMI, kg/m^2^	23.41 ± 3.35	22.08 ± 3.13	24.23 ± 3.23	<0.001
WL%	3.23 (0.00, 6.37)	6.14 (4.17, 9.47)	0.00 (0.00, 3.86)	<0.001
NRS-2002 ≥ 3, *n* (%)	286 (65.00)	150 (89.82)	136 (49.82)	<0.001
CCI score, *n* (%)				0.755
0	333 (75.68)	125 (74.85)	208 (76.19)	
1	75 (17.05)	31 (18.56)	44 (16.12)	
≥2	32 (7.27)	11 (6.59)	21 (7.69)	
ECOG PS score, *n* (%)				<0.001
0	256 (58.18)	66 (39.52)	190 (69.60)	
1	131 (29.77)	64 (38.32)	67 (24.54)	
≥2	53 (12.05)	37 (22.16)	16 (5.86)	
TNM stage, *n* (%)				<0.001
I	107 (24.32)	17 (10.18)	90 (32.97)	
II	84 (19.09)	36 (21.56)	48 (17.58)	
III	189 (42.95)	78 (46.71)	111 (40.66)	
IV	60 (13.64)	36 (21.56)	24 (8.79)	
Anemia, *n* (%)	188 (42.73)	102 (61.08)	86 (31.50)	<0.001
Hypoproteinemia, *n* (%)	135 (30.68)	79 (47.31)	56 (20.51)	<0.001
CRP > 5 mg/L, *n* (%)	99 (22.50)	70 (41.92)	29 (10.62)	<0.001
Anorexia, n (%)	165 (37.50)	132 (79.04)	33 (12.09)	<0.001
Low HGS, *n* (%)	82 (18.64)	67 (40.12)	15 (5.49)	<0.001
NLR	2.47 (1.89, 3.50)	3.17 (2.16, 4.33)	2.29 (1.73, 3.00)	<0.001
ALI	34.31 (23.91, 47.99)	25.89 (16.56, 35.95)	40.43 (29.58, 52.63)	<0.001
SII	515.80 (335.05, 803.30)	675.77 (415.07, 1162.91)	464.12 (299.17, 675.00)	<0.001
SIRI	0.92 (0.60, 1.51)	1.17 (0.75, 2.19)	0.85 (0.54, 1.22)	<0.001
AISI	190.48 (106.20, 337.54)	259.00 (151.86, 528.63)	160.94 (96.80, 280.80)	<0.001
IPI	0.06 (0.03, 0.31)	0.22 (0.04, 0.99)	0.05 (0.03, 0.13)	<0.001
IBI	2.28 (1.25, 11.06)	7.44 (1.69, 33.86)	1.82 (1.12, 4.96)	<0.001

AISI, aggregate index of systemic inflammation; ALI, advanced lung cancer inflammation index; AWGC, Asian Working Group for Cachexia; BMI, body mass index; CCI, Charlson Comorbidity Index; CRP, C-reactive protein; ECOG PS, Eastern cooperative oncology group performance status; HGS, handgrip strength; IBI, inflammatory burden index; IPI, inflammatory prognostic index; NLR, neutrophil-to-lymphocyte ratio; SII, systemic immune-inflammation index; SIRI, systemic inflammatory response index; TNM, tumor–node–metastasis; WL%, percentage of weight loss.

Patients with cachexia had lower BMI (22.08 ± 3.13 vs. 24.23 ± 3.23; *p* < 0.001) and higher WL% [6.14 (4.17–9.47) vs. 0.00 (0.00–3.86); *p* < 0.001] compared with those without cachexia. They also had a higher prevalence of hypoalbuminemia (47.31% vs. 20.51%; *p* < 0.001), elevated CRP levels (41.92% vs. 10.62%; *p* < 0.001), anemia (61.08% vs. 31.50%; *p* < 0.001), anorexia (79.04% vs. 12.09%; *p* < 0.001), and low HGS (40.12% vs. 5.49%; *p* < 0.001). Additionally, patients with cachexia had higher proportions of poor ECOG performance status and advanced TNM stages (*p* < 0.001). However, no significant differences were found in terms of age (*p* = 0.424), CCI score (*p* = 0.755) or sex distribution (*p* = 0.652).

### 3.2 Correlations between systemic inflammatory biomarkers and weight loss percentage

**Figure 2:**
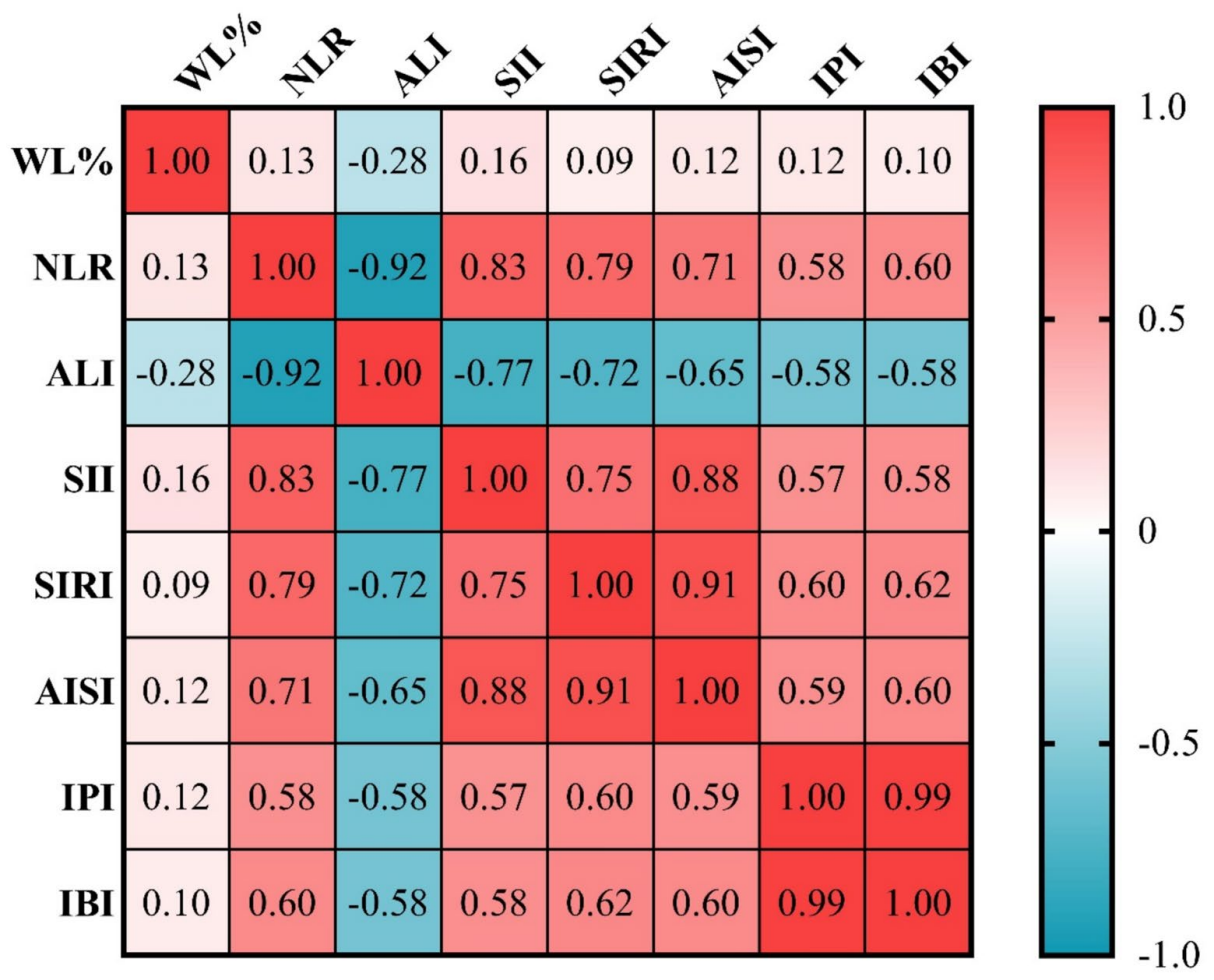
The correlations between WL% and systemic inflammatory biomarkers according to Spearman’s correlation analysis. AISI, aggregate index of systemic inflammation; ALI, advanced lung cancer inflammation index; IBI, inflammatory burden index; IPI, inflammatory prognostic index; NLR, neutrophil-to-lymphocyte ratio; SII, systemic immune-inflammation index; SIRI, systemic inflammatory response index; WL%, percentage of weight loss.

We employed Spearman’s correlation analyses to investigate the correlations between systemic inflammatory biomarkers and WL% in patients with GC. As depicted in Figure 2, the WL% had a significant positive correlation with the NLR (*r* = 0.13, *p* = 0.005), SII (*r* = 0.16, *p* = 0.001), AISI (*r* = 0.12, *p* = 0.009), IPI (*r* = 0.12, *p* = 0.013), IBI (*r* = 0.10, *p* = 0.038), and a negative correlation with the ALI (*r* = −0.28, *p* < 0.001). There was no significant correlation found between the SIRI and the WL% (*r* = 0.09, *p* = 0.063).

### 3.3 Association between systemic inflammatory biomarkers and cancer cachexia

**Figure 3:**
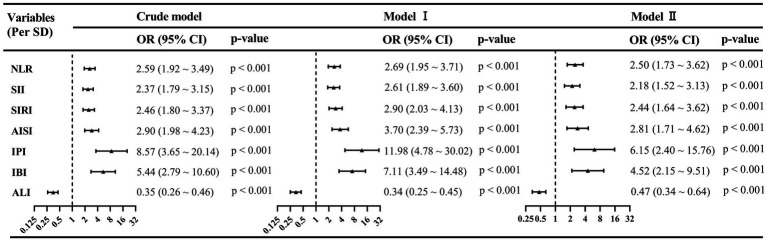
Association between systemic inflammation markers and cancer cachexia based on univariate and multivariate logistic regression analysis. Crude model: unadjusted; Model I: adjusted for age, sex, and BMI; Model II: adjusted for age, sex, BMI, CCI, NRS-2002 score ≥ 3, ECOG PS, TNM stage, hypoproteinemia, and anemia. AISI, aggregate index of systemic inflammation; ALI, advanced lung cancer inflammation index; BMI, body mass index; CCI, Charlson Comorbidity Index; CI, confidence interval; ECOG PS, Eastern Cooperative Oncology Group performance status; OR, odds ratio; IBI, inflammatory burden index; IPI, inflammatory prognostic index; NLR, neutrophil-to-lymphocyte ratio; SD, standard deviation; SII, systemic immune-inflammation index; SIRI, systemic inflammatory response index; TNM, tumor–node–metastasis.

As presented in Table 1, in patients with cachexia, systemic inflammatory biomarkers, including NLR [3.17 (2.16–4.33) vs. 2.29 (1.73–3.00), *p* < 0.001], SII [675.77 (415.07–1162.91) vs. 464.12 (299.17–675.00), *p* < 0.001], SIRI [1.17 (0.75–2.19) vs. 0.85 (0.54–1.22), p < 0.001], AISI [259.00 (151.86–528.63) vs. 160.94 (96.80–280.80), *p* < 0.001], IPI [0.22 (0.04–0.99) vs. 0.05 (0.03–0.13), *p* < 0.001], and IBI [7.44 (1.69–33.86) vs. 1.82 (1.12–4.96), *p* < 0.001] were significantly higher compared to those without cachexia. Moreover, ALI was significantly lower in cachexia patients [25.89 (16.56–35.95)] than in those without cachexia [40.43 (29.58–52.63), *p* < 0.001]. As shown in Figure 3, univariate and multivariate logistic regression models were utilized to evaluate the association between these systemic inflammation biomarkers and cancer cachexia. The results showed that all of these systemic inflammation biomarkers were significantly associated with cancer cachexia in the Crude Model and Model I (all *p* < 0.001). After fully adjusting for potential confounding variables in Model II, for each SD increase in NLR, SII, SIRI, AISI, IPI, IBI, and ALI, the adjusted OR with 95% CI was 2.50 (1.73–3.62), 2.18 (1.52–3.13), 2.44 (1.64–3.62), 2.81 (1.71–4.62), 6.15 (2.40–15.76), 4.52 (2.15–9.51), and 0.47 (0.34–0.64), respectively (all *p* < 0.001).

### 3.4 Evaluations of systemic inflammatory biomarkers using ROC curve

**Figure 4:**
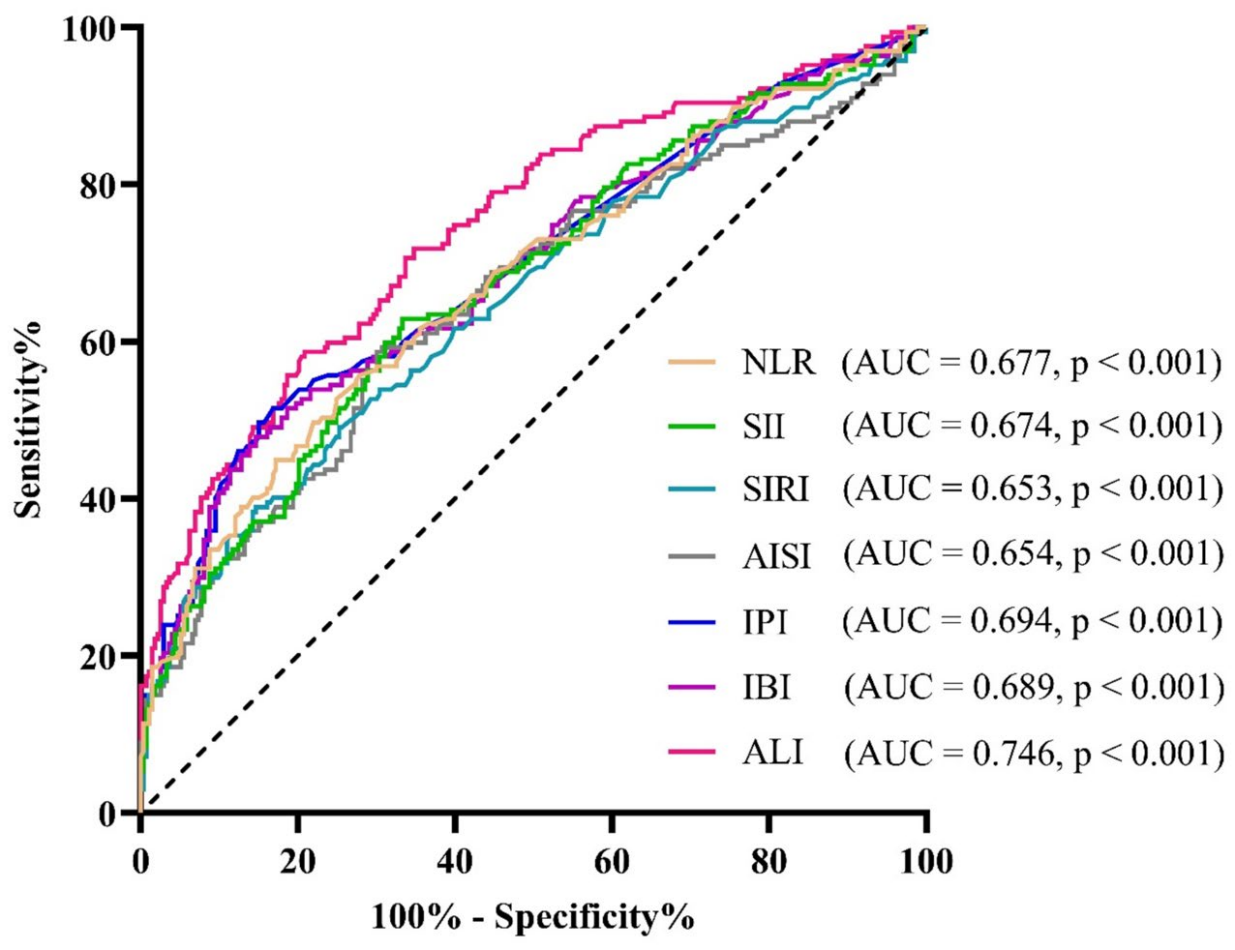
The receiver operating characteristic (ROC) curve for the prediction of cancer cachexia. AISI, aggregate index of systemic inflammation; ALI, advanced lung cancer inflammation index; AUC, area under the curve; IBI, inflammatory burden index; IPI, inflammatory prognostic index; NLR, neutrophil-to-lymphocyte ratio; SII, systemic immune-inflammation index; SIRI, systemic inflammatory response index.

As shown in Figure 4, ROC curves were conducted to determine the AUC, sensitivity, specificity, and optimal cut-off values of the systemic inflammatory biomarkers for identifying cancer cachexia. The AUCs and 95%CI for each systemic inflammatory biomarker were presented as follows: 0.746 (0.698–0.794) for ALI, 0.677 (0.624–0.730) for NLR, 0.674 (0.622–0.727) for SII, 0.653 (0.599–0.707) for SIRI, 0.654 (0.600–0.709) for AISI, 0.694 (0.642–0.747) for IPI, and 0.689 (0.637–0.742) for IBI. Table 2 showed the details of the optimal cutoff values, specificity, and sensitivity. According to the Delong test comparing the AUCs of systemic inflammatory biomarkers with ALI as a reference, the results showed that the AUC of ALI was significantly greater than those of other systemic inflammatory biomarkers (all *p* < 0.05).

**Table 2:** AUCs of systemic inflammatory biomarkers for identifying cancer cachexia.

Variables	ROC curve analysis					Delong test	
	AUC (95% CI)	*p*-value	Cut-off value	Sensitivity (%)	Specificity (%)	*Z*-value	*p*-value
ALI	0.746 (0.698–0.794)	< 0.001	29.06	58.7	79.1	reference	
NLR	0.677 (0.624–0.730)	< 0.001	2.93	55.7	72.5	6.380	< 0.001
SII	0.674 (0.622–0.727)	< 0.001	581.18	62.9	66.7	3.593	< 0.001
SIRI	0.653 (0.599–0.707)	< 0.001	1.57	38.9	85.3	4.531	< 0.001
AISI	0.654 (0.600–0.709)	< 0.001	231.60	58.7	70.0	3.846	< 0.001
IPI	0.694 (0.642–0.747)	< 0.001	0.24	47.9	85.0	2.079	0.038
IBI	0.689 (0.637–0.742)	< 0.001	9.67	47.9	85.3	2.298	0.022

AISI, aggregate index of systemic inflammation; ALI, advanced lung cancer inflammation index; AUC, area under the curve; IBI, inflammatory burden index; IPI, inflammatory prognostic index; NLR, neutrophil-to-lymphocyte ratio; ROC, receiver operating characteristic; SII, systemic immune-inflammation index; SIRI, systemic inflammatory response index; TNM, tumor–node–metastasis.

## 4 Discussion

As far as we know, this is one of the few studies exploring the relationship between systemic inflammation biomarkers and cancer cachexia. In this study, we comprehensively evaluated the predictive potential of NLR and NLR-related systemic inflammatory biomarkers (SII, SIRI, AISI, IPI, IBI, and ALI) for cancer cachexia. Our results showed that the NLR, SII, SIRI, AISI, IPI, and IBI were significantly elevated, while ALI was significantly decreased in GC patients with cachexia. After fully adjusting for potential confounding variables, all these systemic inflammation biomarkers were significantly associated with cancer cachexia. According to the AUC values obtained from the ROC curves, ALI showed the highest AUC with a significant statistical difference, suggesting that it may be a more effective predictor than other systemic inflammatory biomarkers in identifying patients with cancer cachexia.

Ongoing loss of body weight and skeletal muscle are key characteristics of cancer cachexia. Based on the international consensus diagnostic criteria for cancer cachexia, the weight loss and low muscle mass are crucial diagnostic criteria (1). Numerous studies have explored the relationship between inflammation and muscle metabolism, elucidating the underlying mechanisms of this process (24, 25). The direct mechanism through which inflammatory cytokines induce skeletal muscle wasting involves the activation of specific receptors within muscle tissue, resulting in the suppression of muscle protein synthesis, enhanced catabolic activity via the ubiquitin-proteasome pathway and autophagy, and impaired myogenesis (25). A systematic review and meta-analysis conducted by Tuttle et al. demonstrated that elevated levels of circulating inflammatory cytokines are correlated with reduced skeletal muscle strength and muscle mass (10). Furthermore, Abbass et al. (26) confirmed a consistent association between reduced skeletal muscle mass and measures of systemic inflammatory response, including NLR, CRP and albumin levels in patients with different types of cancer. Additionally, according to an international multi-cohort study, Martin et al. further verified that elevated systemic inflammation levels are independently linked to involuntary weight loss (27). Specifically for GC, gastric tumor cells and tumor-associated stromal cells can secrete a panel of pro-inflammatory cytokines that predispose to GC, including IL-6, TNF-α, and transforming growth factor-*β* (TGF-β). These cytokines not only promote tumor proliferation and invasion but also directly induce skeletal muscle protein degradation by activating the ubiquitin-proteasome pathway and inhibiting myoblast differentiation, which is a core pathological process of cachexia in GC patients (28). Furthermore, GC often disrupts the gastric mucosal barrier, leading to bacterial translocation into the circulation (29). This process triggers the excessive secretion of pro-inflammatory cytokines (such as IL-1β and IL-6) and further increases systemic inflammation markers, forming a positive feedback loop that accelerates muscle wasting (3). Moreover, the anatomical location of gastric tumors frequently causes early satiety, mechanical obstruction, and impaired nutrient absorption, creating a synergistic effect with inflammation-driven metabolic dysregulation. In the present study, we found that involuntary weight loss was positively correlated with systemic inflammation biomarkers, such as NLR, SII, AISI, IPI, and IBI, while it was negatively correlated with ALI. These results, combined with GC-specific inflammatory mechanisms, confirm that systemic inflammation plays a pivotal role in weight and skeletal muscle loss among GC patients.

In clinical practice, the NLR is a commonly used systemic inflammatory biomarker because it can be easily obtained through routine blood tests. In recent years, the NLR has emerged as a significant prognostic indicator in various solid tumors, such as colorectal cancer (30), liver cancer (31), ovarian cancer (32), breast cancer (33), and GC (34). Furthermore, NLR-related systemic inflammatory biomarkers, including SII, SIRI, AISI, IPI, IBI, and ALI, have also received increasing attention in clinical settings. Elevated IPI has been reported to be associated with poorer clinical outcomes in patients with metastatic colorectal cancer and GC (17, 35). In patients with esophageal cancer, a higher AISI score was associated with a poorer prognosis (16). A high IBI has been identified as an independent high-risk factor for cancer patients, significantly impacting physical condition, malnutrition, cachexia, and clinical outcomes (18). Furthermore, recent meta-analyses have demonstrated that elevated pre-treatment SII, SIRI, ALI levels are significantly correlated with adverse clinical characteristics and poorer overall survival in GC patients (15, 19, 36, 37). However, the relationship between these systemic inflammatory biomarkers and cancer cachexia has not been fully elucidated. Our findings further indicated that these NLR and NLR-related systemic inflammatory biomarkers were significantly associated with cancer cachexia after adjusting for all potential confounding factors. Therefore, as convenient and cost-effective indicators for assessing the status of systemic inflammation, these biomarkers can be used for the early risk stratification of cancer patients. This, in turn, facilitates the optimization of treatment strategies, including prioritizing nutritional intervention, optimizing anti-tumor treatment regimens, and implementing anti-inflammatory targeted interventions.

Early screening for cancer cachexia is crucial for developing timely interventions to mitigate its adverse clinical outcomes, particularly prior to the onset of the refractory phase. Chen et al. carried out a comparative analysis of the four most frequently used nutritional screening tools, namely the Malnutrition Universal Screening Tool (MUST), the NRS-2002, the Malnutrition Screening Tool (MST), and the Short Nutritional Assessment Questionnaire (SNAQ), for identifying cachexia in patients with curable GC. Their results indicated that MST was the most effective tool for identifying cancer cachexia (38). Nevertheless, it should be noted that these nutritional screening tools possess certain subjectivity and complexity, necessitating operation and interpretation by professionals. In the current study, we further utilized the ROC curve analysis and DeLong tests to compare the predictive performance of these systemic inflammatory biomarkers for cancer cachexia. The findings of this study show that ALI has the largest AUC compared to the other biomarkers, suggesting its potential as the most predictive biomarker. The ALI was initially developed to evaluate the degree of systemic inflammation in patients with metastatic non-small cell lung cancer (39). It is crucial to emphasize that the difference between ALI and other systemic inflammatory biomarkers is that ALI encompasses not only NLR and albumin related to inflammation but also BMI for evaluating nutritional status. This might be one of the reasons why the ALI demonstrated a superior predictive performance in identifying cancer cachexia compared to other systemic inflammatory biomarkers. Moreover, the ALI can be conveniently calculated using routine hematological and anthropometric measurements commonly collected in clinical practice. As a result, ALI can serve as an objective, simple, and reliable biomarker for the early identification of cancer cachexia, presenting a broad application prospect.

This study has some limitations. Firstly, this study was conducted at a single center with a limited sample size and only enrolled Chinese patients with GC. Additionally, our research lacked an external validation cohort. Therefore, large-scale, multicenter prospective studies are needed to validate these findings. Secondly, this is a cross-sectional study with limitations in inferring causal relationships. It is suggested that future research adopt time-series analysis methods to clarify the causal relationship between the dynamic changes of systemic inflammatory biomarkers and the progression of cancer cachexia. Finally, this study solely included the systemic inflammatory biomarkers measured at admission and did not assess their longitudinal changes. It is necessary to conduct dynamic monitoring at multiple time points to more comprehensively evaluate the long-term stability of these biomarkers.

## 5 Conclusion

In conclusion, this study demonstrated that NLR and NLR-related systemic inflammatory biomarkers (SII, SIRI, AISI, IPI, IBI, and ALI) were significantly associated with cancer cachexia. Among these biomarkers, ALI exhibited the highest efficacy in identifying patients with cancer cachexia. These objective and easily accessible systemic inflammatory biomarkers may serve as valuable tools for the early detection of cancer cachexia, thereby contributing to the optimization of treatment strategies.

## Data Availability

The raw data supporting the conclusions of this article will be made available by the authors, without undue reservation.
